# Beyond open access

**DOI:** 10.1002/nop2.46

**Published:** 2016-02-25

**Authors:** Roger Watson

*Nursing Open* is an open access, pay to publish journal which is dedicated to the highest standards in open access publishing. We believe that authors should have the opportunity to make their work freely available to others to download and read, with the ensuing benefits that this entails, both for readers and authors. However, open access opens up further possibilities that are not always available to publishing by the more traditional route of pay to view: that of making your work directly available on a wide range of platforms and also promoting your work in the process. Specifically, I am referring to the use of online social media and among the possibilities are Twitter, Facebook, YouTube, blogging and podcasting. By any of these means – and many of these can be interlinked so that entries on one are automatically relayed by the other platform – you can tell the world about your work and add a link which allows people to read it at no cost.

At *Nursing Open*, we help to promote your work via our Twitter site and our blog. Currently, our policy is to promote every paper on Twitter at the point of going online and also to produce a blog entry for each paper and to promote the blog link on Twitter. However, as the journal grows – and it is growing – we may be unable to promote every paper on our blog. This is where authors can help; have you considered writing a blog entry (we can put that up on our site or make a link to yours), recording a YouTube clip or speaking about your work in a podcast? If not, I would strongly urge you to think about it. Using social media is remarkably easy, and making recordings that can be uploaded to YouTube or on a podcast is also remarkably easy and I want to offer some advice here to our authors and readers who may want to promote their work.

## YouTube

The technical aspects of making a YouTube clip are that the files you upload have to be in the .avi format. This is about as technical as it gets. All you need to make a YouTube clip are a recording device, preferably one with a camera so we can see you. A webcam is ideal but other devices not linked to a computer are available. You need to work out how to save your recording and if that can be saved as an .avi file, then your recording is ready to upload. If your recording is saved in another format, for example .mp4, then you will need to convert it. Online, public domain software is easily available and easy to use. Uploading an .avi file to YouTube requires you to have a Google account and when you are logged in and open YouTube the landing page has a tab for uploading videos. I will leave you to explore the rest. If you do not have a Google account and would prefer not to have one then just send us the file – using an online file transfer procedure (e.g. WeTransfer) as these files are large – and we will do the rest.

## Podcasting

Podcasting can be complex if you try to set up your own podcast site, for example, on Google. However, there are free online podcasting facilities allowing you to upload a few podcasts at no cost; if you want to exceed that then the prices are not high. On the other hand, if you do not wish to set up your own podcast site then, again, send us the recording and we will upload it for you. The basic requirement for a podcast is that you can create .mp3 files and most hand held recording devices (Dictaphones) produce .mp3 files. You also need to be able to transfer the file to a computer to attach to email or upload to a file transfer procedure – again, all much easier that it may sound. Alternatively, make a recording using your computer and save in any format and it is then easy for you, or us, to convert to .mp3 format. Again, free software is available online to convert sound files.

Promoting your work on social media is known to be effective in getting it read and increasing citations (Eysenbach 2011, Knight 2014) so this is not just about social networking. In addition, at *Nursing Open*, we keep track of how often articles are mentioned in social media and this is expressed as an Altmetric and you can see these for each article when you view it online. When it comes to promoting your work, I would urge you to be accurate and truthful; on the other hand, I would also advise you not to be shy.
